# Long-read RNA sequencing reveals extensive transcript isoform changes in a patient with IFAP syndrome with a recurrent intronic *MBTPS2* variant

**DOI:** 10.1038/s41439-026-00347-1

**Published:** 2026-04-14

**Authors:** Toshihiko Iwaki, Yosuke Nishio, Sachiyo Takagi, Keiichiro Fujii, Masanori Fujimoto, Emi Sato, Yuji Nakamura, Daisuke Ieda, Yutaka Negishi, Ayako Hattori, Tomoo Ogi, Shinji Saitoh

**Affiliations:** 1 Department of Pediatrics and Neonatology, Nagoya City University Graduate School of Medical Sciences, Nagoya, Japan; 2 Department of Genetics, Research Institute of Environmental Medicine, Nagoya University, Nagoya, Japan; 3 Department of Geriatric and Environmental Dermatology, Nagoya City University Graduate School of Medical Sciences, Nagoya, Japan; 4 Department of Pathology and Molecular Diagnostics, Nagoya City University Graduate School of Medical Sciences, Nagoya, Japan; 5 Department of Pediatrics, Nagoya City University East Medical Center, Nagoya, Japan

**Keywords:** Neurodevelopmental disorders, Disease genetics

## Abstract

Hemizygous pathogenic variants in *MBTPS2* located at Xp22.12 cause IFAP syndrome, which is characterized by the triad of ichthyosis, alopecia and photophobia. Here we identified a hemizygous intronic variant in *MBTPS2* (NM_015884: c.970+5G>A) in a patient with alopecia, follicular keratinization, developmental delay and epilepsy which were compatible with IFAP syndrome. The variant has been reported to cause skipping of 20 bases in exon 7, leading to the premature termination codon. To further investigate the consequence of the variant, we performed long-read RNA sequencing using RNA extracted from fibroblasts cultured from the patient’s skin. Long-read RNA sequencing revealed skipping of exons 6 and 7, in addition to the previously reported 20-base skipping in exon 7. Furthermore, the expression of normal transcripts, as observed in healthy skin, was markedly reduced. Long-read RNA sequencing is a powerful tool for simultaneously identifying diverse transcript isoforms, making it highly valuable for elucidating the effects of splice-altering variants.

## Discovering splice variants in patients with IFAP syndrome

The study investigates the impact of a recurrent intronic variant in the MBTPS2 gene, which is crucial for cholesterol regulation and stress response, linked to IFAP syndrome. Researchers used long-read RNA sequencing on fibroblasts from a patient with keratosis pilaris, alopecia, developmental delay and epilepsy. This method revealed significant transcript alterations, including skipping of exons 6 and 7, and a reduction in normal transcript expression. The findings contrast with previous studies that identified only a 20-base skipping in exon 7, highlighting the variant’s complex effects on transcript diversity. These transcript changes may contribute to the patient’s mild clinical symptoms, such as ichthyosis and developmental delay. The study underscores the utility of long-read RNA sequencing in uncovering transcript variations caused by intronic variants, suggesting its potential for broader applications in genetic research.

This summary was initially drafted using artificial intelligence, then revised and fact-checked by the author.

## Introduction

The membrane-bound transcription factor protease site-2 gene (*MBTPS2*) is located on the X chromosome and encodes a site-2 protease (S2P). S2P is a membrane-embedded zinc metalloprotease that cleaves and activates several signaling and regulatory proteins from the membrane. S2P plays an important role in the regulation of cholesterol homeostasis and the endoplasmic reticulum stress response^1^.

Ichthyosis follicularis, alopecia and photophobia (IFAP) syndrome is a triad of ichthyosis, alopecia and photophobia, with the first case of ichthyosis follicularis associated with atrichia reported by MacLeod in 1909^2^. Other symptoms include short stature, intellectual disability and seizures, and severe cases may be complicated by BRESHECK syndrome. Currently, *MBTPS2* (Xp22.12) and *SREBF1* (17p11.2) have been reported as pathogenic genes.

We experienced a case of keratosis pilaris, alopecia, developmental delay and epilepsy, and identified a recurrent intronic variant in *MBTPS2*, leading to a diagnosis of IFAP syndrome. In a previous study, Chen et al.^3^ revealed that the *MBTPS2* variant caused 20-base skipping in exon 7, leading to the premature termination codon, with the aberrant transcription confirmed by a minigene assay.

In this study, we performed long-read RNA sequencing (RNA-seq) using RNA sextracted from fibroblasts cultured from the patient’s skin to further investigate the consequence of the variant.

## Materials and methods

### Ethics declaration

This study was approved by the institutional review board of Nagoya City University Graduate School of Medical Sciences (#70-00-00200). Written informed consent was obtained from the parents of the patient for publication of this report.

### Skin biopsy and cell culture

A skin biopsy was performed when the patient was 3 years and 6 months of age. Fibroblasts were established in culture from the skin biopsy specimens. In addition, a battery of dermatopathological tests were conducted.

### Whole-exome sequencing and Sanger sequencing validation

Trio-based whole-exome sequencing was performed at 3 years of age using genomic DNA extracted from the blood of the patient, father and mother through the Initiative on Rare and Undiagnosed Diseases (IRUD) project.^4^ The identified variant was confirmed by PCR amplification and Sanger sequencing with primer #4 (*MBTPS2*-exon7-2F: 5′ CTTTTTGTGGGAGACCTT 3′) and primer #5 (*MBTPS2*-exon7-2R: 5′ CCCGGCTATGATTTAATAC 3′).

### Long-read RNA-seq

Total RNA was extracted from fibroblasts cultured from the patient’s skin and from control fibroblasts. Long-read RNA-seq was performed using the PacBio platform. In brief, full-length cDNA was synthesized using the Iso-Seq Express 2.0 kit (Pacific Biosciences), following the manufacturer’s instructions. SMRTbell libraries were constructed using the Kinnex full-length RNA kit (Pacific Biosciences). The barcoded cDNA was concatenated and converted into circular templates. Libraries were sequenced on the PacBio Revio system. HiFi reads were processed with SMRT Link v12.0, including demultiplexing with lima and identification of full-length nonchimeric reads using isoseq refine. The resulting flnc.bam files were used for downstream transcript analysis.

### Transcript annotation and quantification with TALON

The flnc.bam file was aligned to the GRCh38 reference genome using pbmm2 (v1.17.0). The resulting BAM files were processed with the samtools calmd command to add MD tags, and were then converted to SAM format. To assess internal priming, the reads were labeled using talon_label_reads (TALON v6.01). Transcript annotation was performed using TALON, with labeled reads registered to a database initialized with the GENCODE v44 GTF annotation. High-confidence isoforms were identified using talon_filter_transcripts, applying a minimum read count threshold of 5. Transcript lists were generated separately for patient and control datasets, then merged to produce a unified set of filtered transcripts. A final GTF was created using talon_create_GTF, and transcript abundances were quantified with talon_abundance.

### RT-PCR

Reverse transcription polymerase chain reaction (RT-PCR) was performed using primer #1 (*MBTPS2*-RT-F: 5′ AGCAGCTATTAGGGAACA 3′) spanning exon 4 and exon 5 and primer #3 (*MBTPS2*-RT-R2: 5′ TAACCATCCAGAGAGTCCCA 3′) located in exon 11. Reverse transcription was performed with SuperScript IV First-Strand Synthesis System (Invitrogen, cat. no. 18091050) to create cDNA. RT-PCR products were extracted using the gel extraction kit, and Sanger sequencing was performed. Electrophoresis was performed on a 3% agarose gel at 100 V for 30 min. Toyobo’s Loading Quick 100 bp DNA Ladder was used as a molecular size marker.

## Results

### Case presentation

**Fig. 1:**
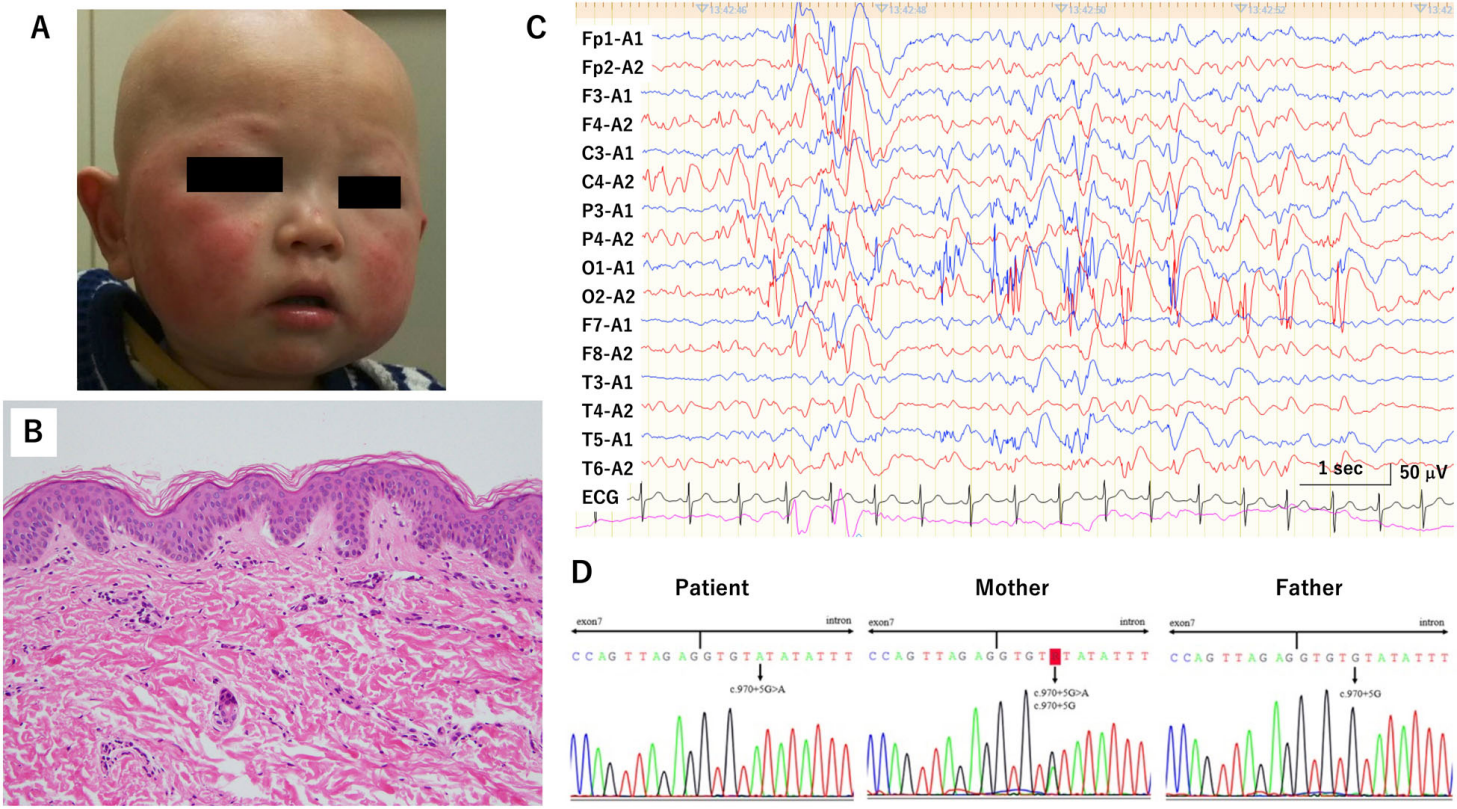
Clinical presentation and genetic diagnosis of the patient. **A** Clinical photograph of the patient at 6 months of age. Hair on the head was present at birth, but both scalp and eyebrows became hairless by 6 months of age. **B** Histological findings from the skin biopsy. Strong enlargement of the epidermis to the upper dermis. Mild hyperkeratosis was observed in the epidermis. The layered structure of the epidermis (cornified layer, granular layer, spinous layer, basal layer) was maintained. Mild lymphocytic infiltration was observed around blood vessels in the upper dermis. **C** Electroencephalography showed diffuse spike-and-slow wave complexes. **D** Sanger sequencing for the patient and parents. A variant in intron 5 was found in the patient (hemizygous) and in the mother (heterozygous).

The patient described in this report was a 3-year-old boy who was born at term with a birth weight of 2465 g (−1.8 s.d.). No family history of similar clinical features was noted. Delayed motor development was observed, with a late turnover at 8 months and independent walking at 2 years and 7 months. No language delays were observed. His medical history showed hair on his head at birth, but hair loss began gradually around 4 months of age, and by 6 months of age, his eyebrows and head had become hairless (Fig. 1A). He was referred to our hospital’s dermatology department at 7 months of age because of dermatitis all over his body. At 1 year and 1 month of age, his dermatitis worsened, and antihistamine medication was added. The patient’s skin continued to develop impetigo, erythematous papules, eczema and dryness, with repeated exacerbations and remissions, resulting in a chronic course of the disease. At the age of 1 year and 10 months, he developed myoclonic seizures, which gradually increased up to 20 times a day. Electroencephalography showed diffuse spike-and-slow wave complexes compatible with myoclonic seizures (Fig. 1C). Oral administration of valproic acid was started, and seizures were suppressed. Blood tests and brain magnetic resonance imaging during the course showed no abnormalities. A skin biopsy was performed at 3 years and 6 months of age. A dermatopathological examination confirmed hyperplasia of keratin, with evidence of ichthyosis (Fig. 1B). No abnormalities were found on ophthalmologic examination.

### Genetic analysis

Whole-exome analysis at 3 years of age revealed a hemizygous intronic variant (NM_015884: c.970+5G>A) in *MBTPS2*, which was present in his mother in a heterozygous fashion. The variant was confirmed by Sanger sequencing (Fig. 1D).

### Long-read RNA-seq

**Fig. 2:**
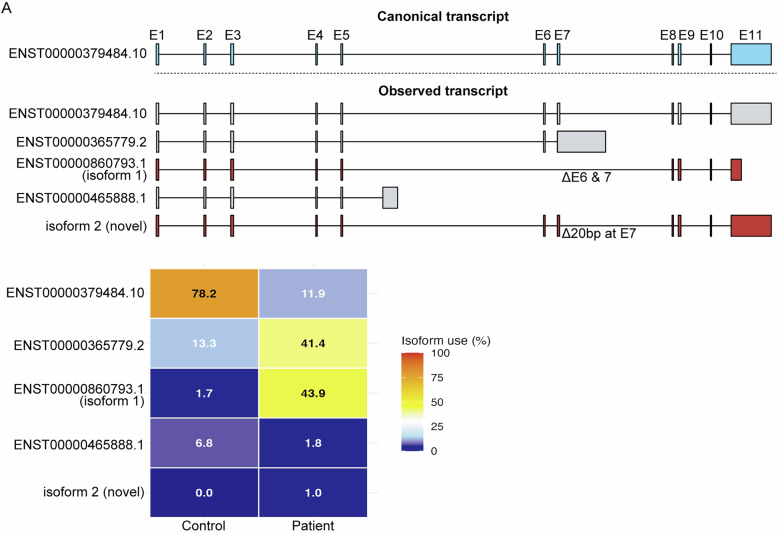
Long-read RNA-seq from patient-derived fibroblasts. Identified variants are shown above. The isoform use rate (%) in the patient and a control are shown by heatmap below. Long-read RNA-seq revealed skipping of exons 6 and 7 (isoform 1), in addition to the previously reported skipping of 20 bases in exon 7 (novel isoform 2) in the patient. Furthermore, the expression of normal transcripts, as observed in healthy skin, was markedly reduced in the patient.

Long-read RNA-seq revealed skipping of exons 6 and 7 (isoform 1) in the patient for 43.9% of transcripts, which was very rare (1.7%) in the control (Fig. 2). The previously reported skipping of 20 bases in exon 7 (isoform 2) was identified only in 1.0% of transcripts, and was not identified in the control (Fig. 2). Furthermore, the expression of normal transcripts was markedly reduced to 11.9% in the patient compared to 78.2% in the control (Fig. 2). In addition, an alternative truncating transcript (ENST00000365779.2) that was observed at a rate of 13.3% in the control was upregulated to 41.4% in the patient.

### RT-PCR

**Fig. 3:**
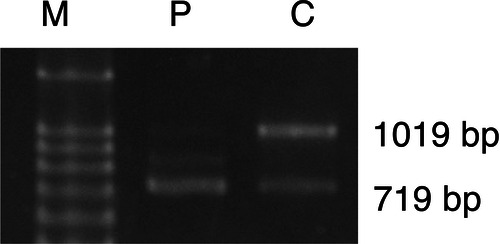
RT-PCR from patient-derived fibroblasts. The 1,019-bp and 719-bp bands correspond to the canonical transcript and the transcript skipping of exons 6 and 7 (isoform 1), respectively. Note the reduced expression of the canonical transcript and the notable increase of the transcript for isoform 1 in the patient. P, patient; C, control; M, size marker (100-bp DNA Ladder).

RT-PCR revealed the two distinct bands of 1,019 bp and 719 bp (Fig. 3). Subsequent Sanger sequencing confirmed that the 1,019-bp and 719-bp bands correspond to the canonical transcript and the transcript skipping of exons 6 and 7 (isoform 1), respectively. It is of note that the normal canonical transcript was markedly reduced while the transcript skipping of exons 6 and 7 (isoform 1) was markedly increased compared with those of the control in the patient.

## Discussion

In mammalian cells, when sterols are depleted, sterol regulatory element binding proteins, which are membrane-bound transcription factors attached to the endoplasmic reticulum, are proteolyzed in two steps by site-1 protease and S2P present in the membrane. The NH2-terminal domain is thereby released from the endoplasmic reticulum membrane. These domains translocate to the nucleus, where they activate genes for cholesterol and fatty acid biosynthesis^1,5–7^. Similar reactions are also observed with other transcription factors, such as activating transcription factor 6 and cyclic AMP-responsive element-binding protein 3-like protein 3^1,8^.

The *MBTPS2* gene encodes a canonical transcript of 4,446 bp comprising 11 exons that encodes the 519 amino acids constituting S2P. Structurally, both the NH2 and COOH terminals of S2P face the cytoplasmic side. Most of S2P is hydrophobic, and eight transmembrane (TM) domains are found in the membrane. There are also three long hydrophilic sequences which project into the lumen. S2P has two zinc-binding motifs with HEIGH sequences in a long hydrophobic region, and aspartic acid 467 of the LDG motif within TM domain 7 is about 300 residues away from the HEIGH sequences. The LDG motif modulates zinc at the active site of S2P^7^. Oeffner et al.^9^ reported that mutations close to a putative hydrophobic intramembrane domain that encompasses the active site LDG motif are more developmentally detrimental than amino-acid exchanges in the amino-terminal portion of the protein. Also, regarding genotype–phenotype correlations, Caengprasath et al.^1^ found that mutations within or near TM domains 5 and 8 contribute to severe symptoms, whereas mutations closer to the NH_2_ or COOH terminus are associated with milder forms of IFAP syndrome.

In this study, we identified the complex combination of transcript alterations by using long-read RNA-seq. Long-read RNA-seq showed that the intronic variant induced skipping of exons 6 and 7, in addition to the previously reported aberrant transcript of 20-base skipping in exon 7. The canonical transcript was markedly reduced, while the alternative truncating transcript (ENST00000365779.2) was upregulated threefold. The transcript change detected by the long-read RNA-seq was confirmed by RT-PCR. Consequently, our results indicate global transcript change by the intronic variant. Notably, the effects of the variant differed among alternative transcripts. The canonical transcript and the transcript skipping of exons 6 and 7 (isoform 1) were markedly affected, whereas the alternative truncating transcript (ENST00000365779.2) was modestly affected. Predicting transcript alterations caused by intronic variants remains challenging; therefore, long-read RNA-seq may provide a valuable approach for obtaining a comprehensive view of transcript changes induced by such variants.

Chen et al.^3^ identified the same intronic variant, and revealed that the variant caused skipping of 20 bases in exon 7, leading to a truncated protein. In their study, they performed a minigene assay and reported that skipping of 20 bases in exon 7 was the major transcript isoform of the variant. However, they used a forward primer located in exon 6 for the RT-PCR assay; thus, skipping of exons 6 and 7 was not detected.

As the detection of aberrant transcripts is dependent on the position of primers for RT-PCR, this method is not ideal for identifying all aberrant transcript isoforms. Meanwhile, long-read RNA-seq is advantageous because it can detect most of the transcript isoforms. In this study, we were able to identify the transcript of skipping of exons 6 and 7, which was not detected in the previous study.

Discrepancies between our findings and those of Chen et al. may also reflect tissue- and cell-type-specific differences in transcript isoform usage. Chen et al. analyzed RNA extracted from leukomonocytes, whereas in this study, long-read RNA-seq and RT-PCR were performed using RNA from patient-derived skin fibroblasts. Furthermore, Chen et al. pointed out that discrepancies between direct cDNA sequencing and minigene assays may arise from differences in experimental systems, such as the use of HEK293T cells and the limited genomic context of the minigene construct. Therefore, discrepancies between the studies may reflect cell-type-specific splicing/isoform abundance.

The skipping of 20 bases in exon 7 leads to the premature termination codon, and thus it is likely to be subject to nonsense-mediated mRNA decay. By contrast, skipping of exons 6 and 7 induces an in-frame deletion of p.Gly224_Arg323del, which is located between the TM domain 5 region to the luminal loop. Although it is not close to the zinc-binding motif, extensive deletions from the TM domain 5 region to the luminal loop may disturb protein function substantially. However, from the skin pathology, the findings of ichthyosis were not strong, and developmental delay and short stature were not severe. These mild clinical presentations might be explained by the presence of residual normal transcript.

In conclusion, we evaluated the consequence of the recurrent intronic variant in *MBTPS2* by long-read RNA-seq and identified diverse transcript isoforms. Long-read RNA-seq is highly valuable for elucidating the effects of splice-altering variants.
